# Problematic Video Game Use and Mental Health among Spanish Adolescents

**DOI:** 10.3390/ijerph20010349

**Published:** 2022-12-26

**Authors:** María Ángeles García-Gil, Fernando Fajardo-Bullón, Irina Rasskin-Gutman, Inmaculada Sánchez-Casado

**Affiliations:** 1 Education Sciences Department, Teacher Training College, University of Extremadura, Avenida de la Universidad, 10003 Cáceres, Spain; 2 Department of Psychology, Faculty of Education and Psychology, University of Extremadura, Avenida de Elvas, 06006 Badajoz, Spain; 3 Department of Psychology, Teacher Training College, University of Extremadura, Avenida de la Universidad, 10003 Cáceres, Spain

**Keywords:** problematic video game use, gaming, adolescents, mental health, VGEQ, Spain

## Abstract

Current scientific evidence points to the importance of studying the link between mental health and problematic video game use in adolescents. The aim of this study was to analyse the correlation between gender and stage of adolescence and problematic video game use, as well as to study the correlation between internalizing and externalizing symptomatology, prosocial behaviour and video game use, and the correlation between video gaming and mental health issues in Spanish 12- to 18-year-olds (M = 14.51; SD = 1.57). For this purpose, the Strengths and Difficulties Questionnaire (SDQ) and the Video Game-Related Experiences Questionnaire (VGEQ) were administered to 1448 secondary school students in Extremadura (Spain), of which 50.8% were women and 49.8% men. The results show that (a) males present more problematic video game use, (b) prosocial behaviour negatively correlates with problematic video game use, and (c) mental health issues and problematic video game use correlate in a negative way. However, the stage of adolescence was not seen to have any effect on the problematic video game use. In conclusion, this study points the effects that problematic video game use can have on the mental health of adolescents and the possible protective role that prosocial behaviours can have on the prevention of problematic video game use.

## 1. Introduction

### 1.1. Analysis of the Mental Health of Adolescents

From early infancy, through interaction in a wide variety of social contexts, a series of physical, socioemotional, and cognitive changes take place in humans which affect their personality, interpersonal relationships, and ultimately their mental health [1]. The World Health Organization (WHO) states that mental health is an integral part of an individual’s health as it represents the entire physical, mental, and social well-being of a person, and not merely the condition of being healthy or disease-free [2]. In addition, it points out that conditions that affect mental health include “mental or neurological problems, and substance use disorders”, which, in turn, can lead to intellectual disabilities, risk of suicide, and/or psychosocial and cognitive difficulties [3].

Current data (published by WHO in 2022) indicate that 13% of the world’s young population (aged 10–19) suffers from some type of mental health condition. This is a considerable risk factor for their future adulthood, since such conditions could potentially turn into mental disorders and diminish their quality of life [2].

These data point to the need for a global approach to identifying, evaluating, and treating mental health problems at an early stage in order to prevent the development of more serious mental conditions in adulthood. This could help to prevent risky behaviour, failed interpersonal relationships, stress, dissatisfaction with life, depression, etc. [1]. To this end, the specific focus of this study was to analyse some of the variables associated with mental health and problematic video game use among the Spanish adolescent population.

There is a correlation between mental health and problematic video game use. Depression and loneliness are linked with problematic video game use. In addition, physical aggression is an antecedent of problematic video game use and anxiety is a consequence of it [4].

When discussing mental health conditions, different taxonomic classifications can be used as a reference, such as the mental disorder classification used in the Diagnostic and Statistical Manual of Mental Disorders (DSM-5) [3], and/or the behavioural and emotional conditions included in the Achenbach System of Empirically Based Assessment manual (ASEBA) [5,6]. Both delve more deeply into mental health issues by differentiating between internalizing and externalizing behaviour or conduct. Internalizing behaviour refers to internal processes, such as anxiety, depression, or the somatization of problems, while externalizing behaviour is considered to be any behaviour that affects the individual’s immediate environment, such as lack of self-control, inability to obey, and aggressiveness, among others [1]. Studying mental health from the point of view of internalizing and externalizing symptomatology is usually recommended when working with a general, non-clinical population [7].

Regarding prevalence, behaviour patterns correlated with emotional difficulties and mental disorders are more common in 10- to 24-year-olds than in older age groups, regardless of gender [8]. With regard to internalizing and externalizing symptomatology in particular, significant differences are found in12- to 19-year-olds, with females reporting more internalizing behaviour and males scoring higher on externalizing behaviour [1,8].

It is also important, however, to look for variables that can modulate the internalizing and externalizing problems in adolescents. To this end, much research has focused on analysing behaviour, particularly prosocial behaviour, in the early stages of childhood and adolescence [8,9,10,11]. These studies reveal that altruistic tendencies can improve the individual’s own sense of well-being as well as helping others. Prosocial behaviour consists of manifesting feelings of solidarity towards others, for example, showing empathy or understanding, which brings about changes in the individual’s personality as a result [11]. Thus, prosocial behaviour develops important emotional responses that affect the individual’s sense of solidarity and capacity to empathise and show concern for others, as well as appreciate the world around them, and this, in turn, has a significant impact on other areas of the individual’s life, such as school [12]. Although studies on the links between prosocial behaviour and problematic video game use are scarce, there are tools in the scientific literature that can measure prosocial behaviour, notably the internationally recognised Strengths and Difficulties Questionnaire (SDQ) [13]. Therefore, a closer examination of the relationship of prosocial behaviour and mental health (specifically internalizing and externalizing symptoms) on a large sample of Spanish adolescents could help to prevent mental health difficulties and other behavioural issues.

### 1.2. Video Gaming in Adolescents and Young People

While prosocial behaviour has a positive correlation with the psychosocial life of teenagers, certain other behaviours, such as problematic video game use, have a negative correlation [14,15,16]. Video games are defined as “electronic entertainment devices that are popular with all age groups, although adolescents tend to use them more intensively” [17]. This intensive use can become problematic and can be linked to internalizing and externalizing symptoms that can ultimately lead to mental disorders. Such cases are mostly due to inappropriate or dysfunctional use [14,15,16]. It should be noted that the concept ‘problematic video game use’ has its own controversies. Although it is true that some people can report problems derived from the time spent playing video games, it is not clear that these problems can be attributed to their use, so it would be premature to consider video game use as a formal diagnosis or category [17]. More research is recommended, because instead of a real problem there may be a moral panic towards the use of video games [18]. Besides, it is also found that more than two out of three players do not manifest any problematic symptoms [19].

Most Spanish ‘gamers’ are aged between 11 and 14, only 20% of which are female [20,21]. While video games are played from the age of 5 to 64, the greatest gender differences in gaming exist among 5- to 14-year-olds, with more males playing than females. These differences disappear, however, in the 30 to 64 age bracket [21]. Regarding the prevalence of video game use in Spain, young males not only play more often, but seem to be at greater risk of developing gaming-related conditions than women. Furthermore, there is a link in male gamers between the use of massively multiplayer online role-playing games (MMORPGs) and anxiety, while problematic video game use among teenage females is much lower [19,20]. Nevertheless, it should be considered that women with more severe gambling and gaming problems, by contrast, tend to present more cognitive biases than men [22,23].

Besides, being young can be another predictor of problematic video game use [24]. Regarding the influence of age, this is not very clear, because while normally the time spent playing video games decreases in late adolescence, if it continues to increase, there is a link on the development of mental health [25]. As a result, these authors posit the need to broaden the study of the link between the ‘age and gender’ variable and problematic video game use to include Spanish 12- to 18-years-olds and to examine the relationship between mental health and problematic video game use.

High rates of problematic video game use have been reported, particularly MMORPGs, where players create avatars and interact virtually with other gamers to accomplish missions and move up levels [20]. Groups or ‘clans’ are formed, making the gaming experience more reinforcing. In other words, MMORPGs involve persistent-state worlds, where the virtual gaming environment “continues to evolve and progress” causing the player’s avatar or clan to change and transform, even when the user has logged off, causing “a kind of compulsion to play” [20]. According to some studies [10,15,26,27,28,29,30], such characteristics can be negative predictors of mental health, especially in adolescents, showing a positive correlation with anxiety, depression, hyperactivity, diminished impulse control, emotional and psychosocial issues, and peer relationship problems [13,14]. While these studies examine the negative correlation between video game use and mental health issues, other studies have shown how video games can be used to improve attentional control, cognitive states, spatial skills, and perception, as well as visual attention [15]. Given this diversity of results, the present study attempts to provide more insight into the link between gender, stage of adolescence and internalizing and externalizing symptomatology, and problematic video game use among teenagers.

### 1.3. This Study

In view of the above, there is a need for new, more probing scientific information on the correlation between gender and stage of adolescence and problematic video game use. This study uses data to shed light on the correlation between teenage mental health (internalizing and externalizing symptomatology) and prosocial behaviour and problematic video game use, an area which has received little attention to date. Understanding this connection should help in the development of suitable, cost-effective educational and health strategies. Finally, the correlation between problematic video game use and the mental health of adolescents is analysed in detail. This should allow appropriate restrictions to be established regarding their use.

Therefore, the present study aims to (1) analyse the correlation between gender and age on problematic video game use and examine its main symptoms, and (2) analyse the correlation between internalizing and externalizing symptoms and prosocial behaviour and video game use and examine how video game use can affect the mental health of Spanish12- to 18-year-olds.

In this sense, the hypotheses of the present study are the following: (1) Males will obtain higher scores in the problematic use of video games compared to females. (2) There will be a negative correlation between prosocial behaviours and problematic video game use and there will be a positive correlation between internalizing and externalizing symptomatology and problematic video game use. (3) There will be a negative correlation between problematic video game use and age.

## 2. Materials and Methods

### 2.1. Participants

A sample of 1448 ESO (Spanish Compulsory/Junior Secondary Education) and Baccalaureate (Pre-University/Senior Secondary Education) students was selected, 708 of whom were male (48.90%), with a mean age of 14.5 years (SD = 1.57). Previously, a complete list of the secondary schools and number of pupils in Caceres and Badajoz was obtained from the local branches of the Extremadura board of education. A random selection of 8 schools was taken from this list. Once access to the school had been granted, the questionnaires were administered to all the years in all the schools. When there was more than one stream per year (e.g., a, b, and c), one was chosen at random; 51.8% of the students in the final sample came from rural areas, while 48.2% came from urban backgrounds. Regarding distribution by year, 22% were in the first year of junior secondary, 21.8% in the second year, 20% in the third year, and 21.9% in the fourth; 14% were in senior secondary school, 53.7% attended state-run schools, while 46.3% attended state-subsidized schools.

The participants’ rights were respected in accordance with the professional code of ethics, including the interviewees’ right to privacy and personal data protection, as per Organic Law 3/2018 of 5 December 2018. All the teachers involved signed an informed consent document before taking part in the survey. Moreover, the parents signed the permission to participate. Finally, the ethical code of the University of Extremadura was adhered to, guaranteeing the impartiality and responsibility of the researchers in the collection and processing of all the data [31].

### 2.2. Instruments

The Strengths and Difficulties Questionnaire (SDQ) [13,32,33]. The SDQ consists of 25 items designed to evaluate emotional and behavioural problems as well as prosocial behaviour in children and adolescents using a self-reported approach. It consists of five self-report scales to measure emotional symptoms, conduct problems, hyperactivity/inattention, peer relationship problems, and prosocial behaviour. For this study, the Total Difficulties score was used together with the externalizing scores, the sum of the hyperactivity scale (sample item: “you are easily distracted, your mind tends to wander”) and the behavioural difficulties scale (sample item: “You often have outbursts or temper tantrums”). Similarly, the internalizing scores were calculated by adding up the emotional symptoms scale (sample item: “You often feel unhappy, down or tearful”), and the peer relationship problems scale (sample item: “You are mainly a loner and tend to play by yourself”). For this study, externalizing and internalizing scores were divided into groups based on cut-off scores for ‘normal’, ‘borderline’, and ‘abnormal’ groups. For both externalizing and internalizing symptoms, 0–7 was considered ‘normal’, 8–9 was considered ‘borderline’, and 10–20 was considered ‘abnormal’ [17].

Following the SDQ guidelines, the total score was obtained by adding the scores of the first four scales: emotional difficulties, conduct problems, hyperactivity, and peer relationship problems; the prosocial scale was not included. The Spanish version of the questionnaire was used in this study [34,35,36]. The prosocial scale included in the main SDQ was used to study the prosocial behaviour. Examples of these questions include “You take other people’s feelings into account” and “I often offer to help people”. Other authors also made use of this instrument, confirming that the SDQ scale is an adequate instrument to determine behavioural problems and emotional symptoms [37]. A Cronbach’s α of 0.729 was obtained, while in the subscales the reliability coefficients are low: internalizing symptoms (Cronbach’s alpha = 0.661); externalizing symptoms (Cronbach’s alpha = 0.669); prosocial (Cronbach’s alpha = 0.565).

The Video Game-Related Experiences Questionnaire (VGEQ) [20]. The VGEQ questionnaire assesses problematic video game use. It comprises 17 questions on items such as concern, denial, increased tolerance, negative affect, reduced activity, loss of control, evasion, and desire to play. The overall reliability of this questionnaire was good (Cronbach’s alpha = 0.89).

Other sociodemographic data were collected via an initial questionnaire to determine the following variables: gender (male and female) and age interval (early adolescence (12–13 years), mid-adolescence (14–17 years), and late adolescence (18 and over) [38].

## 3. Statistical Analysis

As the sample in the study was large (n = 1448) [39,40], parametric tests were performed while ensuring, at the same time, that assumptions of normality and homoscedasticity were also met. Firstly, ANOVA was performed to analyse the gender differences in gaming practices. Robust one-factor ANOVAs were used to examine age, prosocial behaviour, and mental health difficulties (using internalizing and externalizing scales) differences in problematic video game use. The criteria for the creation of groups in the ANOVAs are described in the section on instruments.

In addition, a robust one-factor ANOVA was performed using Welch’s method (heterogeneous variances) to study the problematic video game use differences in the mental health of minors. Three similar-sized groups were formed based on their problematic video game use scores to make sure the sample was balanced. Thus, one group was made up of 495 students with the lowest problematic video game use scores (the ‘never’ group), another group consisted of 483 people with intermediate VGEQ scores (the ‘sometimes’ group), and yet another group comprised 469 people with the highest problematic video game use scores (the ‘a lot’ group). Finally, linear regression was performed (to quantify the relationship between the problematic video game use score and the variables age, gender, externalizing, internalizing) and standardized effect sizes were calculated. Data analysis was performed with the SPSS24 statistical program.

## 4. Results

First, descriptive statistics (mean and standard deviation) were calculated for problematic video game use (M = 24.86, SD = 7.8), as well as for internalizing (M = 4.85, SD = 3.11) and externalizing symptomatology (M = 6.17, SD = 3.13) and prosocial behaviour (M = 8.05, SD = 1.71). Significant higher mean of problematic video game use scores were obtained among the males surveyed used ANOVA. A contrast statistic is obtained (F_w 1, 1445_ = 411.308, *p* = 0.00). The mean of the male group (M = 28.62, SD = 7.97) was significantly higher than that of the female group (M = 21.27, SD = 5.66).

A detailed analysis of the scores on the most relevant VGEQ items revealed that 10.1% of the male students and 0.4% of the female students acknowledged having lost a relationship, job, or academic opportunity because they had prioritized video games.

Furthermore, 20.4% of male students stated that they sometimes lied about how often and how long they spent playing video games. Only 7.5% of female students reported doing so.

Regarding the frequency with which students use video games to block out invasive or unwanted thoughts about their lives, 28.9% of the boys and 12.1% of the girls reported doing so occasionally, 13.8% of the boys and 3.5% of the girls acknowledged doing so quite often, and 6.5% of the boys and 2% of the girls admitted doing it all the time.

Contingency tables were used to examine the frequency of poor or disrupted sleep linked by gaming. The results showed that 13.8% of males and 5.5% of females frequently experienced sleep-related issues associated with video game use. In addition, 2.5% of the males acknowledged that they always or almost always feel agitated when they do not play video games.

The contingency tables also showed that 14% of the students felt the need to spend longer periods of time playing video games in order to feel satisfied and 79.2% of the students recognized that they do not notice time going by when they play video games.

In order to determine differences related to age or stage of adolescence, no statistically significant differences were found by early, mid-, or late adolescence group (*F_w_*_2, 87_ = 2.295, *p* = 0.107). When the ‘mid- ‘and ‘late’ adolescence were put in the same group, still no differences were found between early adolescence and the joint group, so it would seem that the stage of adolescence is not a determinant factor in problematic video game use.

To study the externalizing symptomatology on problematic video game use statistically significant differences in problematic video game use were found based on the internalizing scale scores (*F_w_*_2, 205_ = 5.373, *p* = 0.005). Post hoc multiple comparisons were performed using the Games–Howell test (see Table 1).

When examining the internalizing symptomatology, statistically significant differences were found in the mental health group using the externalizing scale (*F_w_*_2, 374_ = 17.475, *p* < 0.001). Post hoc multiple comparisons were performed using the Games–Howell test as the variances were heterogeneous (see Table 1).

Regarding the examination of prosocial behaviour, statistically significant differences were found in the prosocial behaviour group (*F_w_*_2, 79_ = 7.486, *p* = 0.001). Post hoc multiple comparisons were performed using the Games–Howell test (see Table 1).

Finally, a statistically significant differences were found in the video game use group on mental health issues (*F*_2, 1439_ = 31.852, *p* < 0.001). Post hoc multiple comparisons were performed using Tukey’s test (see Table 2).

A significant and negative correlation was found between age and problematic video game use. A significant correlation was found between being male and a higher problematic video game use. Moreover, there was a significant and positive correlation between externalizing and internalizing symptomatology and problematic video game use. To evaluate the presence of multicollinearity among the predictor variables, the variance inflation factor (VIF) was calculated, obtaining a coefficient close to 1 for all variables, which indicates that there is no relationship between the predictor variables (see Table 3).

Finally, multiple correlation was calculated using gender, age, and externalizing and internalizing symptomatology as predictor variables, with problematic video game use as the criterion variable. A coefficient of multiple determination (R-squared) of 29% was found. This coefficient of multiple determination indicates that 29% of the variance of problematic video game use is explained by the set of predictor variables. Likewise, the coefficient of multiple determination is significant (see Table 4).

## 5. Discussion

The aim of this study was to determine problematic video game use in adolescents by identifying gender and age-related differences among gamers and by analysing the correlation between internalizing, externalizing, and prosocial behaviour and video game use. Finally, an attempt was made to determine how video game use may be linked with the mental health of Spanish adolescents aged 12 to 18 in the Autonomous Community of Extremadura (Spain).

In terms of gender, males scored significantly higher than females in problematic video game use and also in its main associated symptoms. These results coincide with studies carried out by Sans [15] and Gonzálvez et al. [16], where the mean score for negative risks derived from video game use was higher for males than for females. Traditionally, video gaming has been viewed as a form of entertainment enjoyed mainly by males, and according to Barnett et al. [41], it is more highly regarded and popular among men than women. Apart from actual use, differences were also found in the motivating factors that drive males and females to play video games, and this is reflected in the behaviour they present at a later stage. These gender-related differences were also demonstrated in a Spanish study carried out in the Pathological Gambling and other Behavioural Addictions Unit of the Psychiatric Department of the Bellvitge Hospital in L’Hospitalet de Llobregat [22,23]. This study involved a total of 512 patients from 28 health centres and state-funded hospitals. In terms of behaviour, the men in this study presented an evolution towards a more advanced stage of their behaviour difficulties, not only because of the addictive behaviour itself but also because of the need to “escape from the negative feelings associated with gambling and gaming”. Women with more severe gambling and gaming problems, by contrast, tend to present more cognitive biases [22,23]. Thus, in light of these results, we can affirm that, in today’s digital world, the repercussions on teenage mental health are heading in a similar direction [42,43].

Other previous studies [11] indicate that prosocial behaviour can bring about positive changes in the individual’s personality. This finding is consistent with our study, where we observed less problematic video game use among the groups who scored highest in prosocial behaviour (classed as ‘normal’) than among those with lower scores. This kind of prosocial behaviour can be encouraged with a view to achieving a positive correlation between the individual’s behaviour and emotional well-being, and ultimately on their mental health. This approach could help to prevent the so-called ‘online gaming disorder’, which WHO recently added to its International Classification of Diseases [2]. In addition, according to other previous studies, the responsible use of cooperative video games can also help promote prosocial behaviour, improve attentional control, lead to better stress management, and enhance cognitive states and spatial skills [15,44,45,46,47].

Indeed, the relationship between video game use and mental health has been the subject of much research in the post-pandemic era [14,48,49] and the results also show how problematic video game use can correlate with the mental health of adolescents negatively. This is also consistent with the findings of previous studies [14,16] which indicate that inappropriate and problematic video game use can lead to problematics such as sleep disorders, disruptive behaviour, and/or social isolation [44,45,46,47,50]. It seems that the difference in personality of adolescent gamers is rooted in a certain degree of extraversion previously identified by psychopathology experts [51].

However, a gender bias towards males has also appeared in recent studies [25]. Boys tend to devote more leisure time to video games, play them more regularly throughout the week, and do so for longer periods of time. The age differences are not quite as clear, though [25]. While the time spent playing video games normally decreases in late adolescence, when it continues or increases, dysfunctional and maladaptive behaviour does tend to appear and can be linked with the development of positive mental health. However, this study found no age-related differences in video game use among adolescents. The results for the different stages of adolescence were homogenous in this regard. Although the age groups (evolutionary stages of the adolescents) show no difference, the regression analysis shows that as age advances, the problematic use of video games decreases.

Given that video game use is a massively widespread leisure activity, addressing it during adolescence is an important step towards preventing the potential disorders listed in the DSM-5. Therefore, it is essential to monitor the environment and control the amount of time children and teenagers spend on gaming [14,22,23,42,48,49,52,53,54,55]. Regarding gender, males score noticeably higher than females on problematic video game use and its main associated symptoms.

Regarding future research projects, it would be useful to include more sociodemographic characteristics such as social class and access to and availability of video games in the home. Such characteristics may play a part in problematic video game use according to several studies [56,57]. Other factors that may be related to problematic video game use and need to be studied in greater depth are family climate and parental styles [38,58,59]. Regarding the scores obtained in the SDQ, it would be interesting to measure the linked between problematic video game use and academic performance and behaviour and to relate the SDQ scores to the adolescents’ learning outcomes, particularly in these post-pandemic times. In a similar vein, it is important to add that, at Spain’s request, the Council of the European Union has recently included the video game industry in its list of ‘priority creative sectors’ with a view to video games being used as tools for transmitting knowledge, and reinforcing and enriching cultures, so it would seem that their application in the field of education looks promising. Hence the importance of expanding future lines of research which could help establish guidelines for the appropriate use of video games for all age groups and prevent the possible associated risks [33,60].

It is relevant to consider the content of video games, since video games with violent content may affect aggression, increasing antisocial behaviour and negative thoughts and decreasing prosocial behaviour, empathy, and sensitivity to aggression [61].

### Practical Implications

As practical implications, the present study would raise awareness among teachers and families of the effects that problematic video game use can have on the mental health of adolescents and the possible protective role that prosocial behaviours can have on the prevention of problematic video game use. Likewise, it is important to consider the differences in terms of gender and age in the problematic use of video games. However, further research would be necessary to make these assertions.

## 6. Limitations of This Study

One of the main limitations of this study is the use of self-reporting for the gathering of data, given that the social desirability bias could potentially be a drawback. However, the size of the sample and the use of a validated mental health assessment scale offset the likelihood of any such bias. Other versions of the SDQ for collecting data from other informants could also be used with a view to comparing the information provided by the adolescents with that of their parents and/or teachers. It should also be pointed out that this was a cross-sectional study, so causal relationships cannot be established. Likewise, it would have been desirable to include more controls, such as family environment and personality styles. The fact that the prosocial subscale has low reliability is also indicated as a limitation.

## 7. Conclusions and Future Lines of Research

Regarding the research objective of this study, male adolescents were seen to score higher than female adolescents in problematic video game use. The adolescents who scored higher in internalizing and externalizing symptomatology also scored higher in problematic video game use. By contrast, adolescents with higher scores in prosocial behaviour scored lower in problematic video game use. Finally, adolescents with greater mental health difficulties scores also scored higher on problematic video game use. Given these results, it seems important to highlight the fact that males suffer more from problematic video game use and are at equal risk of developing the problem throughout their entire adolescence. Similarly, this study shows that not only does problematic video game use correlated with the mental health of adolescents negatively, but it also shows that there is a negative correlation between prosocial behaviour and the problematic use of video games.

## Figures and Tables

**Table 1:** Problematic video game use means and comparison of means by SDQ internalizing, externalizing and prosocial behaviour scales.

	M (Problematic Video Game Use)	SD	Comparison	MD	*p*	95% CI
Internalizing scale (SDQ)						
Normal	24.6	7.44	Normal-Borderline	−0.1	0.989	[−1.84, 1.63]
Borderline	24.7	8.39	Normal-Abnormal	−2.92	0.004 *	[−5.03, −0.81]
Abnormal	27.52	9.73	Borderline-Abnormal	−2.82	0.032 *	[−5.44, −0.2]
Externalizing scale (SDQ)						
Normal	24.02	7.11	Normal-Borderline	−1.94	0.004 *	[−3.35, −0.52]
Borderline	25.96	8.37	Normal-Abnormal	−3.55	<0.001 *	[−5.12, −1.98]
Abnormal	27.57	9.33	Borderline-Abnormal	−1.61	0.134	[−3.59, 0.36]
Prosocial behaviour (SDQ)						
Normal	24.62	7.68	Normal-Borderline	−3.12	0.012 *	[−5.66, −0.58]
Borderline	27.73	8.04	Normal-Abnormal	−3.37	0.029 *	[−6.45, −0.29]
Abnormal	27.98	9.27	Borderline-Abnormal	−0.25	0.987	[−4.13, 3.64]

SDQ = Strengths and Difficulties Questionnaire, M = mean, SD = standard deviation, MD = mean differences, *p* = p-Games–Howell value, * Significant with *p* value < 0.05.

**Table 2:** SDQ means and means comparison by video game use.

Problematic Video Game Use Score Intervals	M (SDQ)	SD	Comparison	MD	*p*	95% CI
Never	7.81	4.08	Never-sometimes	0.46	0.174	[−0.14, 1.07]
Sometimes	7.35	3.89	Never-a lot	−1.54	0.001 *	[−2.15, −0.93]
A lot	9.35	4.15	Sometimes-A lot	−2	0.001 *	[−2.62, −1.39]

M = Mean, SDQ = Strengths and Difficulties Questionnaire, SD = standard deviation, MD = mean difference, *p* = p-Tukey value, * Significant with *p* value < 0.05.

**Table 3:** Ordinary least squares: correlation, standard deviation, and *t*-statistic *p*-value.

	Coefficient	Standard Deviation	*t*-Statistic	*p*-Value		VIF-Statistic
Constant	35.34	1.74	20.24	<0.0001	***	
Age	−0.045	0.11	−1.963	<0.0498	**	1.01
Gender	−0.49	0.35	−21.67	<0.0001	***	1.03
Externalizing	0.167	0.06	6.767	<0.0001	***	1.11
Internalizing	0.152	0.062	5.998	<0.0001	***	1.14

** Significant with *p* value < 0.05, *** Significant with *p* value < 0.01, VIF *=* Variance inflation factor. Missing or incomplete observations have been eliminated: (*n* = 1). The dependent variable is the total score on problematic video game use.

**Table 4:** Linear regression model.

Average of the VGEQ	24.86	SD of the VGEQ	7.80
Sum of waste squares	62688.43	SD of the regression	6.59
R-squared	0.29	R-squared corrected	0.29
F(4, 1442)	137.02	*p*-value (F)	0
Log-likelihood	−4779.84	Criterion of Akaike	9569.69
Schwarz Criterion	9596.08	Crit. de Hannan-Quinn	9579.54

VGEQ = Video Game-Related Experiences Questionnaire, SD = standard deviation.

## Data Availability

The datasets are available through the corresponding author on reasonable request.
